# Correlation of genomic alterations assessed by next-generation sequencing (NGS) of tumor tissue DNA and circulating tumor DNA (ctDNA) in metastatic renal cell carcinoma (mRCC): potential clinical implications

**DOI:** 10.18632/oncotarget.16833

**Published:** 2017-04-04

**Authors:** Andrew W. Hahn, David M. Gill, Benjamin Maughan, Archana Agarwal, Lubina Arjyal, Sumati Gupta, Jessica Streeter, Erin Bailey, Sumanta K. Pal, Neeraj Agarwal

**Affiliations:** ^1^ Department of Internal Medicine, University of Utah, Salt Lake City, UT, USA; ^2^ Division of Medical Oncology, Huntsman Cancer Institute, Salt Lake City, UT, USA; ^3^ Department of Pathology, University of Utah, Salt Lake City, UT, USA; ^4^ Department of Pharmacy, Huntsman Cancer Institute, Salt Lake City, UT, USA; ^5^ Department of Oncology, City of Hope Comprehensive Cancer Center, Duarte, CA, USA

**Keywords:** circulating tumor DNA NGS, tumor tissue NGS, correlation, metastatic renal cell carcinoma

## Abstract

**Introduction:**

Tumor tissue and circulating tumor DNA (ctDNA) next-generation sequencing (NGS) testing are frequently performed to detect genomic alterations (GAs) to help guide treatment in metastatic renal cell carcinoma (mRCC), especially after progression on standard systemic therapy. Our objective was to assess if GAs detected by ctDNA NGS are different from those detected by tumor tissue NGS, specifically in patients with mRCC, and if these platforms are interchangeable or complimentary.

**Results:**

When controlling for genes tested by both platforms, the median mutation rate for ctDNA was similar to tissue (median 3.0 vs. 1.0, *p* = 0.14). However, the concordance rate between the two platforms was only 8.6%. When comparing GAs by molecular pathway, GAs in tumor tissue were more common for the DNA repair and epigenetic pathways.

**Materials and Methods:**

Results of NGS testing from tumor tissue and ctDNA from 19 sequential mRCC patients were compared. GAs in each were statistically evaluated using the Wilcoxon signed-rank test. The Fischer's exact test was used to compare the incidence of mutations in selected molecular pathways.

**Conclusions:**

When controlling for genes tested by both platforms, similar number of GAs were detected by both tissue and ctDNA based NGS. However, there was discordance in the type of GAs detected suggesting that ctDNA NGS may be more reflective of dynamic tumor genomic heterogeneity. Hence, these two platforms may be considered complementary to each other, rather than interchangeable, for assessment of tumor GAs to guide selection of targeted clinical trial therapies.

## INTRODUCTION

The treatment landscape of metastatic renal cell carcinoma (mRCC) has dramatically improved in the last decade. Recently three targeted therapies: nivolumab, cabozantinib, and lenvatinib in combination with everolimus were approved in salvage therapy setting of mRCC making a total of ten approved targeted therapies with multiple unique mechanisms of action [1, 2]. Despite approval of this many agents, no biomarkers are used for guiding treatment selection. Instead, sequencing of these agents is largely determined by the design of the registration trials leading to approval of these agents, and/or individual anecdotal experiences and perceptions of the prescribing physicians. Furthermore, many of these newly approved agents in the salvage therapy setting have not been compared head to head in randomized trials, thus making the process of selection of one agent over another arbitrary. Clinical trials attempting to analyze the appropriate sequence of targeted therapies have produced heterogeneous data of modest clinical utility [3–5]. A recent retrospective cohort from the International Metastatic Renal Cell Carcinoma Database Consortium (IMDC) of 4800 patients from 25 centers found that 6 targeted therapies (4 VEGF-TKIs and 2 mTOR inhibitors) were used in at least 9% of patients in the third-line setting [6]. The number of patients treated in salvage therapy setting is expected to increase with approval of more efficacious agents.

Due to the aforementioned challenges, next-generation sequencing (NGS) techniques, such as FoundationOne or Guardant360, are frequently performed in the clinic to guide treatment selection, especially in third line or later setting. FoundationOne is a NGS platform that analyzes tumor tissue and characterizes 315 somatic and germline cancer-associated genes (supplementary document 1) [7]. Guardant360 is a NGS platform that uses circulating tumor DNA (ctDNA) to characterize 72 somatic cancer-associated genes (supplementary document 1) [8]. Both modalities are readily available in the clinic, and are often used interchangeably to guide treatment decisions. Limited data exists comparing genomic alterations (GAs) detected by tissue versus ctDNA-based NGS platforms in various solid tumors [9, 10]. This correlation is particularly relevant as a recent report on nine randomly selected patients with solid tumors showed discordance in tissue versus ctDNA GAs profile [9]. No such data has been reported from a correlation focusing specifically on mRCC patients. The purpose of our study is to compare the type and number of GAs detected by tumor tissue and ctDNA NGS specifically in patients with mRCC.

## RESULTS

Nineteen sequential patients with mRCC who had both ctDNA and tumor tissue NGS testing were included. Baseline characteristics are mentioned in Table 1. Median age at diagnosis of mRCC was 54 years and 57.9% of the cohort was female. IMDC risk category was intermediate for 57.9% (11/19) and poor for 21.1% (4/19). The mean time between tumor tissue and ctDNA NGS was 22 months (range 0–70 months). 3 of 19 patients received treatment between their tissue and ctDNA NGS tests. Table 2 lists the specific GAs detected when controlling for the 68 mutations analyzed by both platforms. For the controlled analysis, the median mutation rate for tissue was 3.0 (range 1–8) and ctDNA is 1.0 (range 0–11) (*p* = 0.14). While median mutation rates detected are similar in the matched cohort, the concordance rate was only 8.6%. From Table 2, we performed a further analysis of the concordance rate for patients with ≤ 6 month between tumor tissue and ctDNA NGS (*n* = 8) and ≥ 6 months (*n* = 11). For the ≤ 6 months group, the concordance rate was 11.4%. For the ≥ 6 months group, the concordance rate was 7.8%.

**Table 1:** Baseline characteristics

Age at Diagnosis, Median (95% CI)	54 (25–72)
Gender, Female	11 (57.9%)
ECOG PS	
0—1	16 (84.2%)
>2	3 (15.8%)
Nephrectomy Performed	15 (78.9%)
Clear cell histology	13 (68.4%)
IMDC Risk Category	
Favorable	4 (21.1%)
Intermediate	11 (57.9%)
Poor	4 (21.1%)
LDH U/L, Median (95% CI)	188 (96.0–4913.0)
Metastatic Disease	
Lung	11 (57.9%)
Bones	8 (42.1%)
Lymph Nodes	5 (26.3%)
CNS	1 (5.3%)
Other	4 (21.1%)

**Table 2:** Concordance for only genes analyzed by both tissue and ctDNA NGS testing

Pt	TISSUE BASED NGS PROFILE	# mutations	ctDNA BASED NGS PROFILE	# mutations
**1**	PIK3CA, PTEN, NF1	3	CCND1 (0.1%)	1
**2**	TP53 G105S, VHL L153P, TP53 L25fs*20, CDKN2A, TERT, BRCA2	6	TP53 G105S(0.2%), VHL L153P (0.5%), TP53 R110C (0.5%), TP53 G245S (0.2%)	4
**3**	NF1, TP53, ATM, BRCA1, BRCA2, GNAS, HNF1A, MAP2K2	8	EGFR (0.4%)	1
**4**	NTRK1	1	BRAF, PDGFRA, KIT, CDK6, EGFR, CCND2, MET, CDK4, KRAS, CCNE1, CCND1	11
**5**	ALK, TP53	2	PIK3CA (0.4%)	1
**6**	TP53 T155N, ATM, NTRK3	3	TP53 T155N (14.2%), RB1 (18.0%), PDGFRA	3
**7**	ARID1A	1	FGFR2 (0.4%), JAK2 (0.3%), CDH1 (0.1%), TP53 (0.1%)	4
**8**	VHL, BRCA2, TERT	3	None	0
**9**	VHL, ARID1A, BRCA2, RET	4	None	0
**10**	RB1, TERT, TP53	3	None	0
**11**	PTEN, RB1, TP53, BRCA1	4	None	0
**12**	VHL L89P	1	VHL L89P(3.9%), TP53 (1.7%), MET (0.2%), CDH1 (0.1%)	4
**13**	VHL L135, TP53, CDKN2A, PTEN, CCNE1	5	VHL L135(0.2%), NRAS (0.2%)	2
**14**	TSC1, TP53, MTOR	3	AR (0.2%), PIK3CA (0.2%), STK11 (0.2%), ERBB2 (0.1%), NOTCH1 (0.1%), ARID1A (0.1%	6
**15**	VHL, MTOR, MAP3K	3	None	0
**16**	NF1	1	None	0
**17**	TP53 H193R, RAF T310S, VHL, BRCA2	4	TP53 H193R(0.3%), RAF T310S (0.3%)	2
**18**	VHL P86T, CDKN2A, NTRK1	3	VHL P86T(0.9%)	1
**19**	VHL, ALK, ARID1A	3	GNAS (0.5%)	1
**Tot**		61		41

Legend: Mutations detected by ctDNA are indicated by a percent after the gene. Genes without a percent are amplifications. The percentage listed is the percent of ctDNA that gene encompasses.

Since FoundationOne analyzes 315 genes compared to 72 genes for Guardant360, we also compared all GAs detected by both platforms. The mutations identified by each platform can be found in Supplementary Table 1. In this analysis, the median mutation rate for tissue NGS was 10.0 and for ctDNA was 2.2. The Wilcoxon signed-rank test confirmed a significant difference for total mutations per patient between the two platforms (*p* < 0.0001).

Table 3 shows mutations identified in any of the 5 pre-defined mutation pathways: DNA repair, cell cycle regulation, PI3K, epigenetics, or angiogenesis for each patient in NGS of either tissue or ctDNA. This table includes mutations in any of the 315 genes analyzed by FoundationOne and the 72 genes interrogated by Guardant360. Using the Fischer's exact test, a significant difference was seen between the two platforms for DNA repair (tissue 12/19 vs. ctDNA 0/19, *p* = 0.0001) and the epigenetics pathway (tissue 10/19 vs. ctDNA 0/19, *p* = 0.0004). In the legend of Table 3, genes tested by ctDNA NGS are underlined. For the DNA repair pathway, *BRCA 1/2* were tested by both platforms. 6 patients had a *BRCA* alteration detected by tissue NGS, while none of these patients were *BRCA* positive on ctDNA. For the epigenetics pathway, the genes analyzed were only detected by tissue NGS and not by ctDNA testing. The other pathways demonstrated no significant difference in mutations between tissue and ctDNA NGS.

**Table 3:** Tumor genomic abnormalities as detected by tissue versus ctDNA NGS testing

Pt	DNA repair		Cell cycle regulation		PI3K		Epigenetics		Angiogenesis	
	Tissue	ctDNA	Tissue	ctDNA	Tissue	ctDNA	Tissue	ctDNA	Tissue	ctDNA
1	X		X	X	X		X			
2	X		X	X	X		X		X	X
3	X		X				X			
4			X	X			X			
5			X			X				
6	X		X	X						
7				X	X		X			
8	X						X		X	
9	X				X		X			
10	X		X							
11	X		X		X					
12	X			X	X				X	X
13			X		X				X	X
14	X					X	X			
15									X	
16	X		X							
17	X		X	X			X		X	
18			X						X	X
19					X		X		X	
TOTAL	12	0	12	7	8	2	10	0	8	4
P VAL	0.0001		0.19		0.06		0.0004		0.30	

Legend:

DNA repair includes: BAP1, POLE, FANCD2, EMSY, FANCF, POLD1, BARD1, PMS2, BRCA1, BRCA2, PALB2.

Cell cycle regulation: BTG1, CCND1, CCND2, CCND3, CDKN2A, CDKN2B, CCNE1, TP53.

PI3K: PREX2, RICTOR, PIK3CA, RPTOR, PTEN.

Epigenetics: MLL2, SETD2, DOT1L, CHD2, KDM6A, DNMT2A.

Angiogenesis: VHL, FLT4.

Genes tested by ctDNA are underlined. All genes were tested by tissue NGS.

## DISCUSSION

To our knowledge, this is the first report on correlation of GAs detected by the ctDNA versus tumor tissue NGS focusing on mRCC patients. ctDNA NGS offers the advantage of decreased risk for testing and the ease of repetitive testing over tumor tissue NGS, which may be reasons to order ctDNA over tumor tissue NGS. However, our findings show low concordance of identified GAs between the two platforms despite similar median mutations rates (Table 2). When comparing all genes analyzed by both platforms, the mean mutation rates become markedly different (Supplementary Table 1). When evaluated by mutational pathways, significant differences between clinically relevant GAs were observed for the DNA repair pathway (Table 3). Our results are consistent with two prior studies evaluating ctDNA to tissue based NGS in “other” unselected solid tumors [9, 10]. In a recent study by Kuderer et al., GAs identified by ctDNA and tissue NGS were compared for 9 patients with a variety of solid tumors. A total of 45 mutations were detected with only 22% concordance between the two platforms [9]. Similarly, the study by Chae et al. compared 28 patients with a variety of advanced malignancies and found 11.8% concordance for GAs detected by either platform, which is more consistent with our findings [10].

It is unknown currently if these differences in the GAs profile between ctDNA NGS and tumor tissue NGS are relevant in prediction of treatment effect. For example, in our study, tumor tissue NGS revealed higher number of GAs in DNA repair genes. Some data suggests that this finding may be clinically relevant. Dung Le et al. demonstrated that in colorectal cancer, DNA mismatch repair-deficiency predicted treatment response to nivolumab therapy [11]. They conducted a phase 2 clinical trial using a PD-1 inhibitor in 41 patients with metastatic colorectal cancer with or without mismatch repair deficiency. They found that patients whose tumors had higher somatic mutation burdens experienced longer progression-free survival when treated with a PD-1 inhibitor. More appropriate assessment of GAs in the DNA repair pathway might be essential prior to selection of immune checkpoint therapy over other targeted therapies in mRCC.

For the angiogenesis pathway, the difference between tumor tissue and ctDNA NGS is not of statistical significance. Yet, it is still worth considering the discordance observed for *VHL* in the mRCC patient population. *VHL* is a truncal mutation in mRCC and thought to be conserved across all subclones [12]. Yet, the concordance rate for *VHL* is only 50% (4/8). Temporal or spatial heterogeneity does not provide an adequate explanation for the discordance observed in this important gene. Finally, mutations in *SETD2*, *PBRM1*, and *BAP1* are thought to have potential prognostic significance in mRCC [13]. Mutations in these genes were detected for some patients in tumor tissue (Supplementary Table 1); however, Guardant360 does not currently test for these genes. These differences could become relevant to clinicians considering using liquid biopsy to guide treatment of mRCC.

Differences in the GAs profile among these two NGS platforms could have resulted due to tumor heterogeneity. Indeed, tumor heterogeneity has been demonstrated frequently in cancers, including in renal cell carcinoma [14]. In 2012, a landmark study of 4 patients with mRCC showed marked intratumoral genomic heterogeneity by tissue based NGS for multiple tumor suppressor genes relevant to RCC, including SETD2, MTOR, KDM5C and PTEN [14]. This study compared the genetic landscape between the primary tumor and metastatic sites and found that 65% of somatic mutations were not detectable in every region of the primary tumor and metastases. It raises the possibility that the anatomic location of the tissue selected for tumor tissue NGS testing in our study has clinical implications. A follow-up study by the authors showed that most driver mutations in RCC are sub-clonal and only VHL aberrations and chromosome 3p loss were conserved events across all clones [12].

The sensitivity of ctDNA testing in mRCC might also be a cause of the discordance in our study. Bettagowda et al. recently analyzed ctDNA levels in patients with a variety of metastatic malignancies, including but not limited to mRCC [15]. mRCC was one of the malignancies found to have a relatively low yield of ctDNA, i.e. < 50% of patients had measurable ctDNA). Interestingly, Pal, et al. presented an oral abstract at the 2017 Genitourinary Cancers Symposium that found detectable ctDNA in 80% of the 270 mRCC tested [16]. While both studies looked at patients with mRCC, Bettagowda, et al. only analyzed 5 patients with mRCC while Pal, et al. reviewed ctDNA data on over 250 patients.

The comparison between tumor tissue and ctDNA has demonstrated marked variability in the published literature with some studies having high concordance and others with wide variability between platforms [17, 18]. One study compared ctDNA to tissue based NGS for EGFR mutations, instead of a comprehensive panel, in patients with non-small cell lung cancer and found a concordance rate of 88% [17]. Of note, the concordance rate reported was for patients with and without EGFR mutations present. However, other studies comparing more comprehensive GA profiles between the ctDNA and tumor tissue testing platforms in unselected solid tumors have demonstrated a high level of discordance between cfDNA and tissue based platforms, which is more consistent with our findings [9, 10]. While liquid biopsies have many attractive attributes including ease and safety, and ability to provide more updated information on tumor GAs over the tissue biopsies, there are also limitations associated with this approach. We anticipate the appropriate use of these tests for the oncology community will become clearer in the coming years.

Limitations of this study include the relatively small sample size. Second is the lack of correlation with treatment specific outcomes, such as response rates, progression free- and overall survival, with respect to the underlying GAs detected by these two platforms, which was not considered feasible because of relatively small sample size. Furthermore, neither tumor tissue or cfDNA NGS tests have been linked to clinical outcomes in patients with any advanced malignancy [19]. In our cohort, the time between tissue and ctDNA NGS introduces the potential for increasing intra-tumor diversity and is as a limitation. Although, when comparing concordance rates between patients with ≤ 6 months and ≥ 6 months between the two tests, there was minimal difference in concordance rates. These limitations will hopefully be addressed in a larger, prospective study being conducted in this setting (NCT02620527).

## MATERIALS AND METHODS

In this Institutional Review Board approved study where written patient consent was obtained, patients were identified who had mRCC and both tumor tissue and ctDNA NGS performed. NGS of tumor tissue was performed by Foundation Medicine (Cambridge, MA). All tumor tissue samples were from nephrectomy of the primary tumor. No metastatic sites were biopsied. NGS of ctDNA was performed by Guardant360 by their standard collection protocol (Redwood, CA). Per Guardant360's standard collection protocol, blood is collected into two 10 mL Streck tubes in order to obtain 5.0–30.0 ng of DNA. Clinical characteristics were obtained by retrospective chart review for the 19 patients. The data collected included patient and disease characteristics, dates of biopsy and blood sample collection (Table 1).

The specific GAs and total number of aberrations identified by both assays were compared (Supplementary Table 1). We then controlled for the 68 mutations detected by both platforms (Table 2). The total number of aberrations detected was statistically evaluated using the Wilcoxon signed-rank test. Concordance was defined as total number of concordant alterations with the denominator as the total number of mutations detected in patient group [10]. The GAs detected by each modality were then grouped into mutational pathways for further analysis. The pathways used were DNA repair, cell cycle regulation, PI3K, epigenetics, and angiogenesis. The specific aberrations used for each pathway can be found in the legend of Table 3. The Fischer's exact test was used to compare the incidence of mutations in identified mutation pathways.

## CONCLUSIONS

We provide the first report correlating GAs detected by ctDNA versus tumor tissue NGS in the mRCC population. Based on this hypothesis generating data, tumor tissue NGS may detect more GAs, and ctDNA NGS may be more reflective of dynamic tumor genomic heterogeneity. Hence, these two platforms may be considered complementary to each other, rather than interchangeable, for assessment of tumor GAs to guide selection of targeted clinical trial therapies.

## SUPPLEMENTARY MATERIALS FIGURES AND TABLES




