# Supplementary material for: Correlation of genomic alterations assessed by next-generation sequencing (NGS) of tumor tissue DNA and circulating tumor DNA (ctDNA) in metastatic renal cell carcinoma (mRCC): potential clinical implications
Source: Oncotarget. 2017 Apr 4;8(20):33614–20. doi: 10.18632/oncotarget.16833 (PMC5464894; doi:10.18632/oncotarget.16833)
Supplement: Supplementary file 1 [file oncotarget-08-33614-s001.pdf]

Correlation of genomic alterations assessed by next-generation sequencing (NGS) of tumor tissue DNA and circulating tumor DNA (ctDNA) in metastatic renal cell carcinoma (mRCC): potential clinical implications

Supplementary Materials

Supplementary Table 1: Mutations identified by tissue compared to ctDNA NGS

Pt	TISSUE BASED NGS PROFILE	# mutations	ctDNA BASED NGS PROFILE	# mutations
1	PIK3CA, PTEN, BAP1, BRD4, BTG1, CCND3, CHD2, CIC, FAT1, MLL2, NF1, POLE, PREX2, RICTOR, RUNX1T1	15	CCND1	1
2	TP53 G105S, VHL L153P, TP53 L25fs*20, CDKN2A, CDKN2B, SETD2, TERT promoter, BLM, BRCA2, DOT1L, EPHA5, IKBKE, MED12, MLL2, MYCN, PIK3C2b, TGFBR2	17	TP53 G105S, VHL L153P, TP53 R110C, TP53 G245S	4
3	NF1, BAP1, TP53, ARID1B, ATM, BRCA1/2, GNAS, HNF1A, MAP2K2, MLL2, SDHA	11	EGFR	1
4	CDKN2C, CD79A, EPHA5, FAT1, HSP90AA1, KDM6A, NTRK1, RUNX1T1, SPEN, ZBTB2	10	RHOA, BRAF, PDGFRA, KIT, CDK6, EGFR, CCND2, MET, CDK4, KRAS, CCNE1, CCND1	12
5	ALK, EP300, FANCD2, FGFR4, IRS2, PRSS8, TOP2A, TP53, WISP3	9	PIK3CA	1
6	TP53 T155N, EMSY, HGF, ARID1B, ATM, BARD1, BRIP1, MLL, NTRK3, PBRM1, SDHD	11	TP53 T155N, RB1, PDGFRA	3
7	ARID1A, PBRM1, SETD2, EPHA5, ERBB4, MAP3K1, RPTOR	7	FGFR2, JAK2, CDH1, TP53	4
8	VHL, BRCA2, PBRM1, SETD2, TERT, AXL, FANCF, FGFR4, GLI1, POLD1, RANBP2	11	None	0
9	MDM2, DNMT3A, FRS2, PBRM1, SETD2, TET2, VHL, ARID1A, BARD1, BRCA2, EP300, MLL3, PDGFRB, RET, RICTOR, TSC2	16	None	0
10	RB1, TERT, TP53, ARID2, CIC, CREBBP, GATA2, NOTCH2, PALB2, PMS2	10	None	0
11	PTEN, RB1, TP53, BRCA1, CRLF2, ERBB3, MEN1, PALB2, SPTA1	9	None	0
12	VHL L89P, ASXL1, ARID1B, ATRX, BAP1, FLT4, PREX2, RSC1, SNCAIP	9	VHL L89P, TP53, MET, CDH1	4
13	VHL L135, TP53, CDKN2A/B, PTEN, AKT2, AXL, CCNE1	7	VHL L135, NRAS	2
14	TSC1, GATA2, PARK2, TP53, BARD1, CHD2, HSD3B1, MAP3K1, MTOR, PALB2	10	AR, PIK3CA, STK11, ERBB2, NOTCH1, ARID1A	6
15	PBRM1, VHL, MTOR, BCORL1, EPHA5, ERBB3, MAP3K	7	None	0
16	KDMSC, CCND3, FANCD2, NF1, POLE, PRSS8, SPEN	7	None	0
17	TP53 H193R, RAF T310S, VHL, BAP1, BRCA2, CD274, MLL2, NOTCH2, NTRK2	9	TP53 H193R, RAF T310S	2
18	VHL P86T, CDKN2A, PBRM1, CARD11, CBL, NTRK	6	VHL P86T	1
19	PBRM1, SETD2, VHL, ALK, ARID1A, ATRX, BLM, HSP90AA1, PRKDC, RICTOR, PTPN11	11	GNAS	1

Supplementary Document 1: For FoundationOne, 315 cancer related genes interrogated for mutation. See Supplementary_Document_1.
