# Supplementary material for: Correlation of genomic alterations assessed by next-generation sequencing (NGS) of tumor tissue DNA and circulating tumor DNA (ctDNA) in metastatic renal cell carcinoma (mRCC): potential clinical implications
Source: Oncotarget. 2017 Apr 4;8(20):33614–20. doi: 10.18632/oncotarget.16833 (PMC5464894; doi:10.18632/oncotarget.16833)
Supplement: Supplementary file 2 [file oncotarget-08-33614-s002.docx]

Supplementary Document 1: For FoundationOne, 315 cancer related genes interrogated for mutation:

ABL1 ABL2 ACVR1B AKT1 AKT2

AKT3 ALK AMER1 APC AR

ARAF ARFRP ARID1A ARID1B ARID2

ASXL1 ATM ATR ATRX AURKA

AURKB AXIN1 AXL BAP1 BARD1

BCL2 BCL2L1 BCL2L2 BCL6 BCOR

BCOR BCORL1 BLM BRAF BRCA1

BRCA2 BRD4 BRIP1 BTG1 BTK

C11orf30 CARD11 CBFB CBL CCND1

CCND2 CCND3 CCNE1 CD274 CD79A

CD79B CDC73 CDH1 CDK12 CDK4

CDK6 CDKN1A CDKN1B CDKN2A CDKN2B

CDKN2C CEBPA CHD2 CHD4 CHEK1

CHEK2 CIC CREBBP CRKL CRLF2

CSF1R CTCF CTNNA1 CTNNB1 CUL3

CYLD DAXX DDR2 DICER1 DNMT3A

DOTIL EGFR EP300 EPHA3 EPHA5

EPHA7 EPHB1 ERBB1 ERBB2 ERBB3

ERBB4 ERG ERRFI1 ESR1 EZH2

FAM46C FANCA FANCC FANCD2 FANCE

FANCF FANCG FANCL FAS FAT1

FBXW7 FGF10 FGF14 FGF19 FGF23

FGF3 FGF6 FGFR1 FGFR2 FGFR3

FGFR4 FH FLCN FLT1 FLT3

FLT4 FOXL2 FOXP1 FRS2 FUBP1

GABRA6 GATA1 GATA2 GATA3 GATA4

GATA6 GID4 GLI1 GNA11 GNA13

GNAQ GNAS GPR124 GRIN2A GRM3

GSK3B H3F3A HRAS HSD3B1 HSP90AA1

IDH1 IDH2 IGF2 IKBKE IKZF1

IL7R INHBA INPP4B IRF2 IRF4

IRS2 JAK1 JAK2 JAK3 JUN

KAT6A KDM5A KDM5C KDM6A KDR

KEAP1 KEL KIT KLHL6 KMT2A

KMT2C KMT2D KRAS LMO1 LRP1B

LYN LZTR1 MAGI2 MAP2K1 MAP2K2

MAP2K4 MAP3K1 MCL1 MDM2 MDM4

MED12 MEF2B MEN1 MET MITF

MLH1 MPL MRE11A MSH2 MSH6

MTOR MUTYH MYC MYCL MYCN

MYD88 NF1 NF2 NFE2L2 NFKBIA

NKX2-1 NOTCH1 NOTCH2 NOTCH3 NPM1

NRAS NSD1 NTRK1 NTRK2 NTRK3

NUP93 PAK3 PALB2 PARK2 PAX5

PRBM1 PDCD1LG2 PDGFRA PDGFRB PDK1

PIK3C2B PIK3CA PIK3CB PIK3CG PIK3R1

PIK3R2 PLCG2 PMS2 POLD1 POLE

PPP2R1A PDRM1 PREX2 PRKAR1A PRKCI

PRKDC PRSS8 PTCH1 PTEN PTPN11

QKI RAC1 RAD50 RAD51 RAF1

RANBP2 RARA RB1 RBM10 RET

RICTOR RNF43 ROS1 RPTOR RUNX1

RUNX1T1 SDHA SDHB SDHC SDHD

SETD2 SF3B1 SLIT2 SMAD2 SMAD3

SMAD4 SMARCA4 SMARCB1 SMO SNCAIP

SOCS1 SOX10 SOX9 SOX2 SPEN

SPOP SPTA1 SRC STAG2 STAT3

STAT4 STK11 SUFU SYK TAF1

TBX3 TERC TERT TET2 TGFBR2

TNFAIP3 TNFRSF14 TOP1 TOP2A TP53

TSC1 TSC2 TSHR U2AF1 VEGFA

VHL WISP3 WTI XPO1 ZBTB2

ZNF217 ZNF703

For FoundationOne, 28 gene rearrangements:

ALK BCL2 BCR BRAF BRCA1

BRCA2 BRD4 EGFR ETV1 ETV4

ETV5 ETV6 FGFR1 FGFR2 FGFR3

KIT MSH2 MYB MYC NOTCH2

NTRK1 NTRK2 PDGFRA RAF1 RARA

RET ROS1 TMPRSS2

For Guardant360, it looks at 72 point mutations. Of these 72 point mutations, it also analyzes 23 indels, 18 amplifications, and 6 fusion genes. Underlined genes are tested for in Guardant360, but not in FoundationOne

AKT1 ALK APC AR ARAF

ARID1A ATM BRCA1 BRCA2 CCND1

CCND2 CCNE1 CDH1 CDK4 CDK6

CDKN2A CTNNB1 DDR2 EGFR ERBB2

ESR1 EZH2 FBXW7 FGFR1 FGFR2

FGFR3 GATA3 GNA11 GNAQ GNAS

HNF1A HRAS IDH1 IDH2 JAK2

JAK3 KIT KRAS MAP2K1 MAP2K2

MAPK1 MAPK3 MET MLH1 MPL

MTOR MYC NF1 NFE2L2 NOTCH1

NPM1 NRAS NTRK1 NTRK3 PDGFRA

PIK3CA PTEN PTPN11 RAF1 RB1

RET RHEB RHOA RIT1 ROS1

SMAD4 SMO STK11 TERT TP53

TSC1 VHL
